# Decoding the Locus of Covert Visuospatial Attention from EEG Signals

**DOI:** 10.1371/journal.pone.0160304

**Published:** 2016-08-16

**Authors:** Thomas Thiery, Tarek Lajnef, Karim Jerbi, Martin Arguin, Mercedes Aubin, Pierre Jolicoeur

**Affiliations:** 1 Université de Montréal, Montréal, Québec, Canada; 2 Centre de recherche en neuropsychologie et cognition (CERNEC), Montréal, Québec, Canada; 3 International Laboratory for Brain, Music, and Sound Research (BRAMS), Montréal, Québec, Canada; 4 Centre de recherche de l’Institut universitaire de gériatrie de Montréal (CRIUGM), Québec, Canada; 5 LETI Lab Sfax National Engineering School (ENIS), University of Sfax, Sfax, Tunisia; University of Groningen, NETHERLANDS

## Abstract

Visuospatial attention can be deployed to different locations in space independently of ocular fixation, and studies have shown that event-related potential (ERP) components can effectively index whether such covert visuospatial attention is deployed to the left or right visual field. However, it is not clear whether we may obtain a more precise spatial localization of the focus of attention based on the EEG signals during central fixation. In this study, we used a modified Posner cueing task with an endogenous cue to determine the degree to which information in the EEG signal can be used to track visual spatial attention in presentation sequences lasting 200 ms. We used a machine learning classification method to evaluate how well EEG signals discriminate between four different locations of the focus of attention. We then used a multi-class support vector machine (SVM) and a leave-one-out cross-validation framework to evaluate the decoding accuracy (DA). We found that ERP-based features from occipital and parietal regions showed a statistically significant valid prediction of the location of the focus of visuospatial attention (DA = 57%, *p* < .001, chance-level 25%). The mean distance between the predicted and the true focus of attention was 0.62 letter positions, which represented a mean error of 0.55 degrees of visual angle. In addition, ERP responses also successfully predicted whether spatial attention was allocated or not to a given location with an accuracy of 79% (*p* < .001). These findings are discussed in terms of their implications for visuospatial attention decoding and future paths for research are proposed.

## Introduction

According to Desimone and Duncan (1995), processing of visual information is characterized by two distinct phenomena. First, due to the limits of the human cognitive system, there is often more information available than what can be fully processed. When this occurs, there is a competition between various items in view and their associated locations for access to capacity-limited processes. Second, in order to resolve this competition while maximizing the pertinence of the information that will be processed effectively, a selection of relevant items must take place. This selection can be bottom-up (exogenous), driven by the properties of the stimuli, or top-down (endogenous), driven by the goals of the observer; or it can reflect an interaction between these two influences (e.g., Leblanc, Prime, & Jolicoeur, 2008).

To study these phenomena, cognitive psychologists have used experimental tasks designed to investigate the effects of the covert orienting of attention in response to different cue conditions, such as the Posner cueing task (Posner, 1980). In these tasks, each trial begins with an endogenous or exogenous cue that indicates the location of a subsequent target with some probability. Then, a target is presented. The target appears either at the cued location (valid trials) or at an uncued location (invalid trials). Several studies [1–3] have shown that response times are shorter for valid trials compared to invalid trials, in both exogenous and endogenous cueing paradigms. These results, together with equivalent or higher accuracy at cued versus uncued locations, suggest that visual spatial attention is deployed towards the cued location, and that an item benefits from preferential processing at this location.

Moreover, electroencephalography (EEG) is often used to explore the temporal dynamics of brain activity that underlies the performance of such cueing tasks [4–7]. In particular, brain responses directly related to shifts of visuospatial attention can be probed using event-related potentials (ERPs).

Single-trial EEG typically is far too noisy to track attention with current paradigms. To deal with this issue, the standard practice is to average a large number of trials with respect to stimulus onset [8]. This time-domain averaging technique results in an ERP wave time-locked to stimulus processing that usually displays a few distinct peaks at specific electrodes. The amplitude and latency of these ERP peaks are the two main properties that are measured to characterize the involvement of the underlying brain regions in the task at hand [9,10].

In the present study, two ERP components are explored. First, the ERP component N2pc (typically around 200 ms, and “pc” meaning “posterior-contralateral”), maximal at occcipito-lateral electrode sites (PO7/PO8) contralateral to the target, has been linked to selective attention [11–16]. The N2pc component can be isolated from motor and sensory activity by subtracting the electrical activity of electrodes over ipsilateral scalp sites from that measured at the corresponding electrodes contralateral to the visual field of an attended item (e.g., PO7/PO8). Generally, the N2pc component starts around 180 ms post-stimulus onset and lasts approximately 100 ms, but the paradigm can impact the onset and the duration of this component. While Luck and colleagues, initially suggested that the N2pc reflects distractor suppression processes, other authors reported findings suggesting that the N2pc component might reflect enhanced target processing (e.g., Eimer, 1996).

Similar to the N2pc, the sustained posterior contralateral negativity (SPCN), which starts around 300 ms post stimulus onset, also arises at electrode sites contralateral to task-relevant visual items, and has a posterior scalp distribution consistent with activity in the extrastriate visual cortex (McCollough, Machizawa, & Vogel, 2007). Specifically, the SPCN is thought to reflect retention in short-term visual memory [15,17–21]. Interestingly, modulations of SPCN amplitude have been reported in a working memory task, without modulations of the N2pc component, suggesting that the N2pc and the SPCN are two functionally distinct components (Jolicoeur et al., 2008).

In the present study, we extracted the well-studied ERP components N2pc and SPCN and used them as features that served as inputs to a machine-learning classification pipeline. In particular, we used supervised classification, a widely used machine-learning technique where predictive models of group membership are constructed from the training data. In the training set, the labels (classes) associated with the signal features are provided. This results in a trained classifier that can then be applied to naïve data segments (test set). Iteratively changing the way the data is split into training and test sets, results in a cross-validation framework that quantifies classification performance or decoding accuracy (DA). Machine learning methods have been successfully used in a variety of fields, and can deal with high-dimensional data often accompanied by small sample sizes [22] and lately, methods such as support vector machines (SVM) have become increasingly popular for the classification of EEG signals especially in brain–computer interface research [23–25].

Despite the extensive literature on visuospatial attention, little is known about the extent to which ERP components can be used to predict its spatial location. The goal of this study was to address this question by investigating the feasibility of using ERP components to recover the locus of covert visual spatial attention from ERP measures. To do so, we implemented a modified Posner cueing task with endogenous cues to direct attention to one of four locations on either side of a fixation point. In the Posner paradigm, attention shifts have long been characterized as voluntary or automatic depending on the type of cue used (endogenous vs. exogenous, respectively; e.g. Jonides, 1981; Yantis & Jonides, 1984). An abrupt visual onset of a unique stimulus at a peripheral location (exogenous cue) is typically assumed to result in an automatic shift of attention towards its location. In contrast, a symbolic cue (in other words, an endogenous cue), such as an arrow pointing left or right, shown at fixation, is said to produce voluntary shifts of attention towards a particular location. However, peripheral cues may induce voluntary shifts of attention (e.g.[26]) provided that cue onset involves a number of different stimuli displayed at distinct competing locations. In that case, the cueing stimuli must be interpreted and a particular stimulus value (e.g., large checkerboards, instead of small ones) is the one towards which attention must be directed in a way that we assume is voluntary. Given the properties of the cueing stimuli used in the present experiment, we call them endogenous and we assume that the attention shifts produced by the participants were voluntary. This interpretation may be open to discussion, but it does not detract from the conclusion that our present findings indicate a capacity to determine the direction of attention through ERP recordings. A final decision as to whether the attention shifts we measure were indeed voluntary will need to await further research. We used multi-class SVM to evaluate the accuracy with which the locus of attention could be decoded from the EEG signal, and we used the same features to classify attended versus non-attended stimuli.

## Methods and Materials

### Participants

Sixteen healthy participants provided their written and informed consent to participate in this study that was approved by the Ethics Committee of the Faculty of Arts and Science (CERFAS) of the Université de Montréal (Canada). All participants were neurologically normal, and had normal or corrected-to-normal vision. Of the 16 participants, one was excluded due to numerous ocular artifacts (rejection rate of 30% due to many blinks throughout the experiment). Fifteen participants were included in the final analysis (age: *Mean age* = 23.2, 3 males, 1 left-handed).

### Stimuli and Procedure

**Fig 1:**
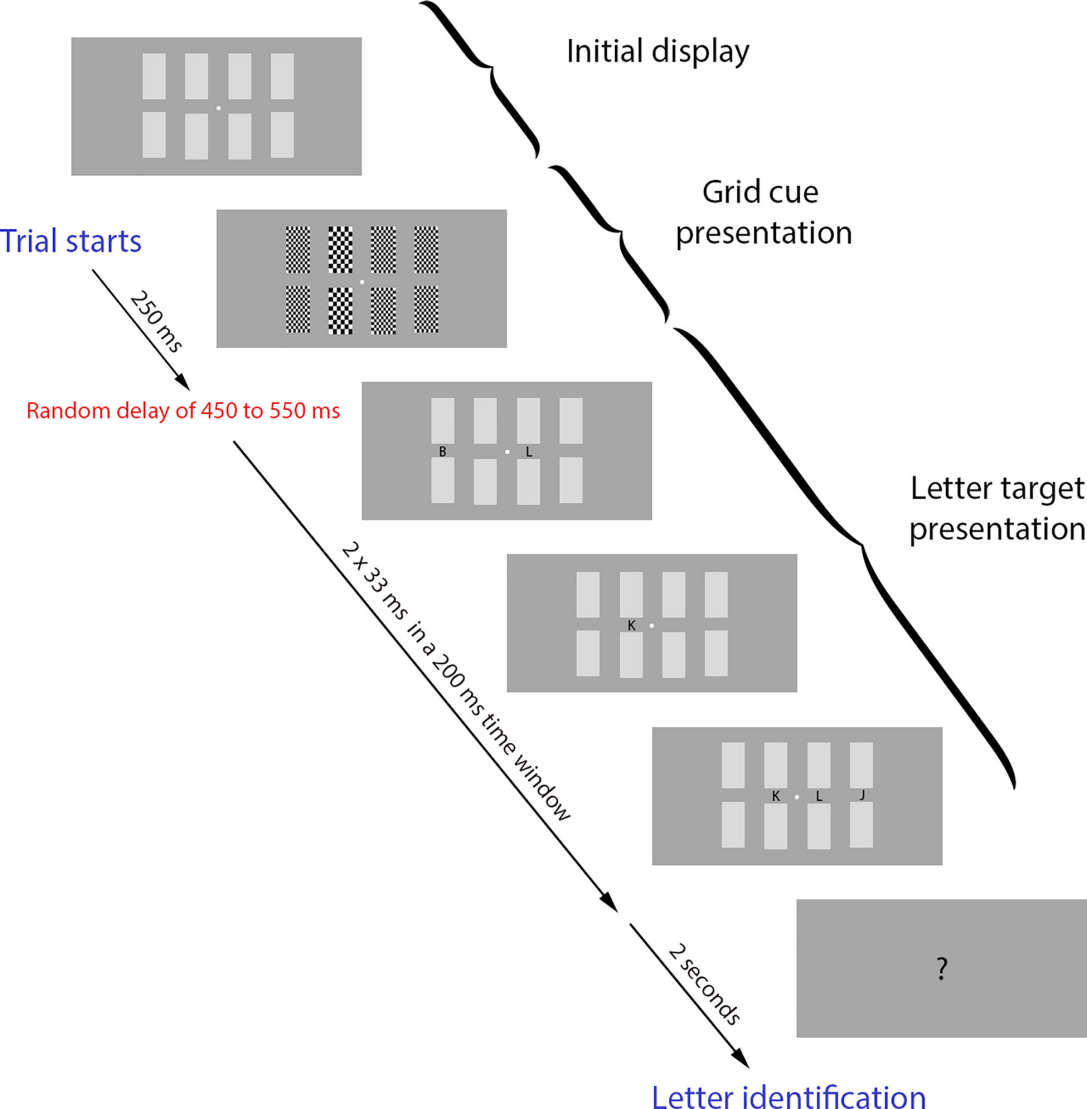
Experiment design (modified Posner task). The grid cues (2^nd^ frame) were presented for 250 ms. Next, each letter was flashed twice (33 ms each time) at random points in time within a 200 ms time window.

Stimuli were presented on a dark grey background on a cathode-ray tube (60 Hz) using the PsychToolbox-3 implemented in Matlab (Brainard, 1997; Pelli, 1997; Kleiner et al., 2007). A central fixation point was presented during the whole duration of the trial, as well as 8 medium grey squares, symmetrically disposed around the fixation point (4 on each side), forming two lines of four rectangles corresponding to the four locations where the cue and the target letter would appear (vertical gap: 2.05°, horizontal gap: 0.88°) (See Fig 1). Each trial began when the participants pressed the space bar on a keyboard placed in front of them. After 100 ms, the white fixation point turned dark gray for a random duration, ranging from 750 to 1000 ms, indicating that the cue was about to appear. At this point, the eight medium-grey rectangles, representing the four locations (See Fig 1), switched to checkered grids for 250 ms. The grids were composed of either finer or coarser elements. The coarser grid cued the participant to attend to that location. The grids had approximately the same mean luminance as the previous grey field in which half of the pixels were black and half were white, and finer and coarser grids had the same mean luminance. Following the offset of the cue, there was a random delay of 450 to 550 ms before the onset of the letter sequence, when four letters appeared (one letter per location). Letters were presented independently (i.e., could occur at the same time, or not, with any letter in the other positions) and they randomly flashed twice, each flash lasting for a duration of 33 ms, with a minimum delay of 17 ms between flashes within a time window of 200 ms. The distribution of the random time windows was uniform. The task was to identify the cued letter after a question mark was displayed on the screen 2 seconds after the end of the 200 ms display period. The participants completed two blocks of 300 trials each (600 trials total), and trials for each of the four cued locations occurred in equal numbers (150 at each location) and in random order.

### EEG acquisition and preprocessing

EEG data from 64 active Ag/AgCl electrodes were recorded using a Biosemi Active Two EEG system. EEG was recorded at a sampling frequency of 512 Hz from electrodes mounted on an elastic cap using the International 10–10 System [27]. Re-referencing of the EEG recording was done offline to the average of the right and left mastoid electrodes. Eye movements and blinks were monitored using horizontal and vertical electrooculography (HEOG/VEOG). A high-pass filter of 0.1 Hz and a low-pass filter of 30 Hz were applied to the EEG signals offline post-recording. The HEOG and VEOG signals were filtered with a high-pass filter of 0.1 Hz and a low-pass filter of 10 Hz. As mentioned above, there were two flashes of each letter, and each one produced a separate ERP. Stimulus-locked ERP events from all trials were epoched in windows ranging from 200 ms pre-stimulus to 800 ms post-stimulus. A baseline correction was performed to the average voltage of the 200 ms pre-stimulus. All epochs containing blinks (VEOG deflection > 50 μV within a time window of 150 ms), eye movements (HEOG deflection > 35 μV within a time window of 300 ms), and other artefacts (signal exceeding ±100 μV) in the EEG signal were excluded from subsequent analysis.

### ERP feature extraction

The feature extraction step consisted of computing the grand average lateralized (Left electrode minus Right electrode) event-related potential (ERP) difference waveforms from 3 electrode pairs (PO7/PO8, O1/O2, and PO3/PO4). This was done by averaging the data with regards to (a) the onset of the attention orientation cue, and (b) the onset of the visual target (letter at the cued location), and further averaging, in each case, the obtained ERP differences over two time windows (early and late, selected on the basis of the grand average waveforms, as detailed below). We chose the time windows used for feature computation based on the ERP literature indicating that the peak of the N2pc component was generally found around 200 ms, and that the SPCN component was a long lasting component starting around 300 ms. We expected to have a better decoding accuracy by choosing time windows corresponding to the peak of the N2pc component and within the duration of the SPCN component, and by choosing channels of interest (PO7/PO8, O1/O2, PO3/PO4) known to be at or near the peak of the N2pc and SPCN scalp distributions (e.g., Jolicoeur, Brisson, & Robitaille, 2008). These features were computed for each of the four cued locations. This provided a total of 12 features (location Cue and Target [2] x electrode-pair [3] x time window [2]) in each of the 15 participants.

#### Cue-locked feature computation

For the cue-locked analysis, we computed the lateralized ERP waves corresponding to each location (1, 2, 3, or 4) for electrodes PO7/PO8, O1/O2, and PO3/PO4 by subtracting the ERP activity measured over the right hemisphere electrode from the ERP activity recorded at the corresponding electrode over the left hemisphere; e.g., PO7 minus PO8. Next, for each location we computed the mean amplitude of the three lateralized ERP measures (PO7/PO8, O1/O2, and PO3/PO4) over two time windows, 170–270 ms and 650–840 ms after cue onset. This provided us with two measures per electrode pair (i.e., 6 features in all). The selection of these time windows was guided by our motivation to have two features (an early and a late one) for lateralized ERP measure. The early time window (170–270 ms after cue onset) is around at the time we expect to see the N2pc component, that usually starts around 180 ms post-stimulus onset and lasts approximately 100 ms. The late time window (650–840 ms after cue onset), was selected so it would likely be in the time interval of the long lasting SPCN component, which starts at about 300 ms. Importantly, we tried to do the same analysis with small differences concerning the selection of time windows (± 20 ms) and the results were not affected in a significant way, suggesting that the results are robust over variations in the precise parameters of the analysis windows.

**Fig 2:**
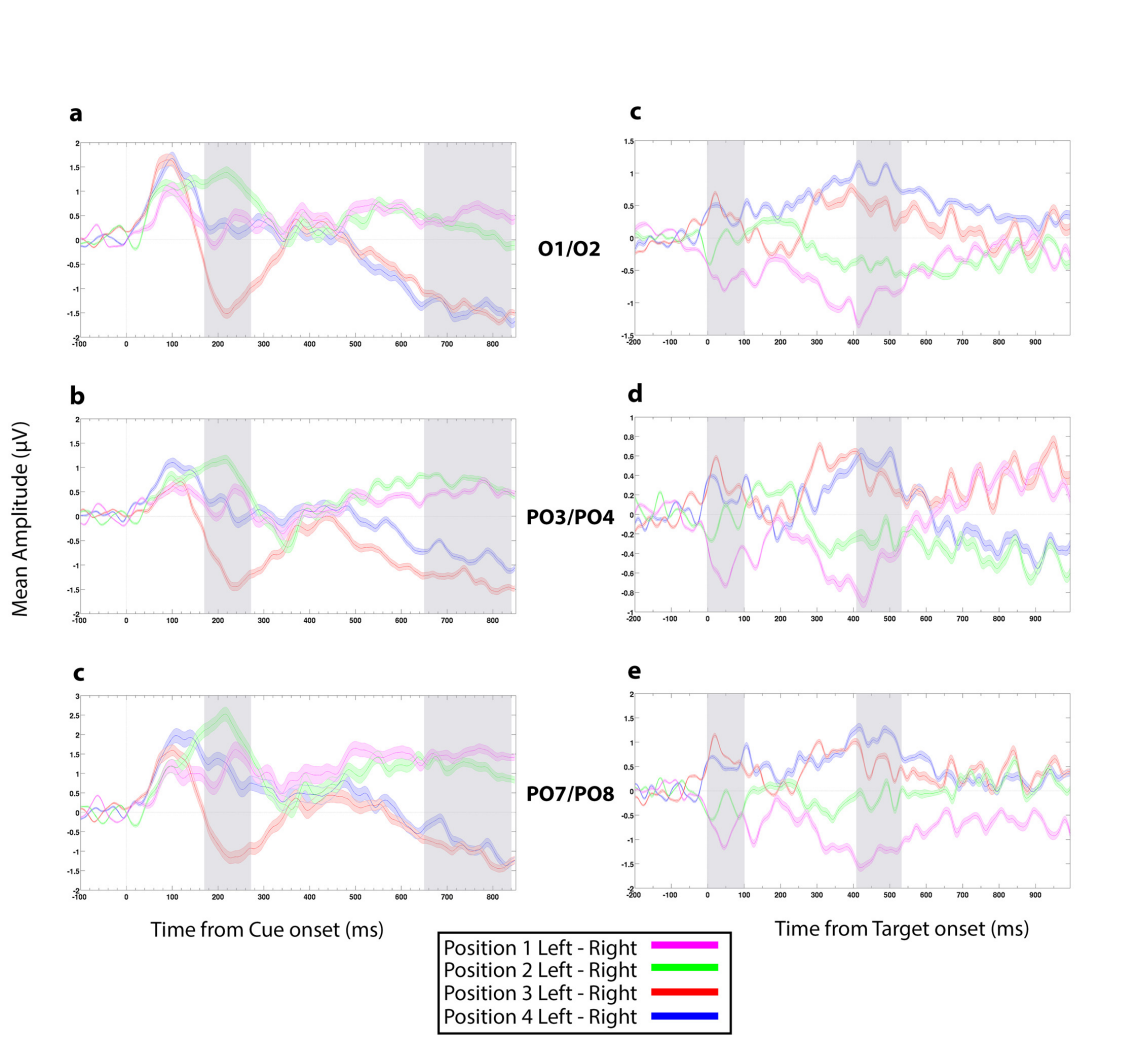
Lateralized ERP waves for electrode pairs PO7/PO8, PO3/PO4, and O1/O2. (a-c) Grand average lateralized (Left electrode minus Right electrode) event-related potential (ERP) difference waveforms, time-locked to the onset of the cue, for the four cue positions (1 to 4 from left to right) for different electrode pairs. (d-f) Same as panels (a-c) but for ERPs locked to the target onset (t = 0 at target letter onset). Note the different scales for d-f. Grey rectangles represent the chosen early and late time windows, which were set to be 170–270 ms and 650–840 ms after cue onset and 0–100 ms and 410–530 ms after target onset. The width of the shaded region around each waveform corresponds to the standard error mean (SEM) across the 15 subjects.

As can be seen in the difference waveforms for the cue-locked left-electrode minus right-electrode difference waves (Fig 2A–2C), follow the patterns expected for N2pc and SPCN for cue-locations 2 and 3, namely a positive difference for the location just left of fixation and a negative difference for the location just right of fixation. This is what we expected if attending to the cued location produced a contralateral negativity. The patterns for the locations further from fixation (locations 1 and 4), were not as clear, in this time window, for reasons we do not understand at the moment and that could be due to the cue or the design of the experiment. The expected contralateral versus ipsilateral patterns were very clear, however, for all locations in the later, SPCN, time window. The expected contralateral versus ipsilateral patterns were very clear, however, for all locations in the later, SPCN, time window. The fact that the patterns of lateralized ERPs were different for different positions in the different time windows, even if not entirely consistent with expectations based on N2pc and SPCN, provides information about which location was cued, which could be used to decode the locus of attention. Unless otherwise stated, these six markers were the cue-locked ERP features we computed for each participant, and that were used to discriminate the four possible locations of the focus of covert attention.

#### Target-locked feature computation

Signal averaging for target letters was relative to the onset of the letter presented at the cued location, but otherwise the target-locked features were computed in the same way as the cue-locked ERPs. Different latencies were used for the early and late time windows, however, which set to 0–100 ms and 410–530 ms after target onset, respectively. The selection of these time windows was also guided by our motivation to have an early feature and a late feature that would correspond to an initial sensory response and a later more cognitive response. For target-locked ERPs, we did not expect to see N2pc or SPCN components because attention should already have been deployed at the cued location at the time of target presentation. Thus, we expected that a stimulus appearing at that location would evoke a stronger response than a stimulus at other locations, and that this effect would likely be lateralized. Often, stimuli appearing at a cued location produce a larger contralateral P1. They also often produce a larger N1, sometimes greater at contralateral electrodes, but also, sometimes larger at ipsilateral electrodes (see Mangun, 1995, and Luck, Fan, & Hillyard, 1993). The waveforms in Fig 2D–2F are consistent with a larger contralateral P1 response (visible here in the 0–100 ms period), followed by a later larger contralateral positivity (which we measured between 410 and 530 ms). Note the particularly early P1 is likely a consequence of averaging over two letter onsets, the second of which could be nearly 200 ms later than the first.

### Machine Learning Analysis

In this study, we applied machine learning to address two distinct classification problems. The first and main question was whether we could use lateralized ERPs to decode the locus of attention out of 4 possible locations (i.e., a 4-class decoding problem). The second question used binary classification to discriminate between the neural responses to attended versus unattended stimuli. In both analyses, we explored a number of classification algorithms including linear-discriminant analysis (LDA), k-nearest-neighbor (KNN), and support vector machine (SVM) with either linear or quadratic (RBF) kernels. The results were very similar across the methods, with slightly better results using quadratic SVMs. Hence, the results presented here were computed using quadratic SVMs.

#### Feature selection

Feature selection was based on the literature of ERP components involved in visual spatial attention (e.g., Jolicoeur, Brisson, & Robitaille, 2008; Mangun 1995). Lateralized ERP waves from electrodes pairs PO7/PO8, PO3/PO4, and O1/O2 (see ERP analysis section) were computed with respect to (i) orientation cue and to (ii) target presentation. These lateralized ERP amplitudes were then averaged over time windows as described in the ERP analysis section above. This led to 12 features (location Cue and Target [2] x electrode-pair [3] x time window [2]), for each of the 15 individuals.

#### Multi-class SVM classification technique

Numerous reports in the literature provide evidence for the high performance of SVM, in particular for high dimensional classification problems [28,29]. SVMs were initially designed for binary classification problems. However, a number of strategies have been proposed to embed SVM classifiers in multi-class decoding frameworks. Two of the most widely used approaches for multi-class SVM classification are the One-Against-All (OAA) and the One-Against-One (OAO) approaches. For the purpose of the current study, we used a dendrogram SVM (DSVM), a decision-tree-based multi-SVM classification that has been explored in the machine learning and computer science literature [30–33], and has been used recently for the automatic classification of sleep stages [34]. In this framework, a binary SVM is trained at each node of the decision-tree and the optimal hierarchical structure of the decision tree is obtained via hierarchical clustering analysis (HCA). Associating decision tree architecture with binary SVMs combines the advantages of the efficient computation of decision trees and the high classification accuracy of SVMs.

#### Evaluation of decoding accuracy via cross-validation

The performance of the proposed classification method was evaluated using a Leave-One-Out (LOO) cross-validation procedure. This procedure is a special case of k-fold cross validation, where all individuals except one are used for training, and the classifier is tested on the data from the omitted participant (i.e., on data for which the classifier was not trained). This procedure is repeated as many times as there were participants, each time leaving a different individual out of the training data. Given that we had 15 participants; the procedure was completed in 15 iterations, with each iteration producing either a correct or an incorrect classification of the untrained, test, data set. For example, a decoding accuracy of 66% would correspond to 10 correct predictions out of 15. The LOO cross-validation method efficiently uses data and provides an asymptotically unbiased estimate of the averaged classification error probability over all possible training sets [35].

## Results

### Behavioral results

All 15 participants were able to perform the letter-identification task without difficulty and had a mean accuracy of 95%.

### ERP analysis and feature extraction

Fig 2 shows the grand average of lateralized cue-locked and target-locked ERP waveforms for the three electrode pairs O1/O2, PO3/PO4, and PO7/PO8, for each of the four cue position (labeled 1 to 4 from left to right). The individual-subject ERPs were used to compute 12 features for each subject, as described in the Method section. As can be seen in Fig 2, the waveforms clearly differentiated the four positions, in the grand averages. The question was whether, and with what spatial accuracy, cue and target locations could be decoded on the basis of individual-subject ERPs

### Decoding the locus of attention using SVM classification

**Fig 3:**
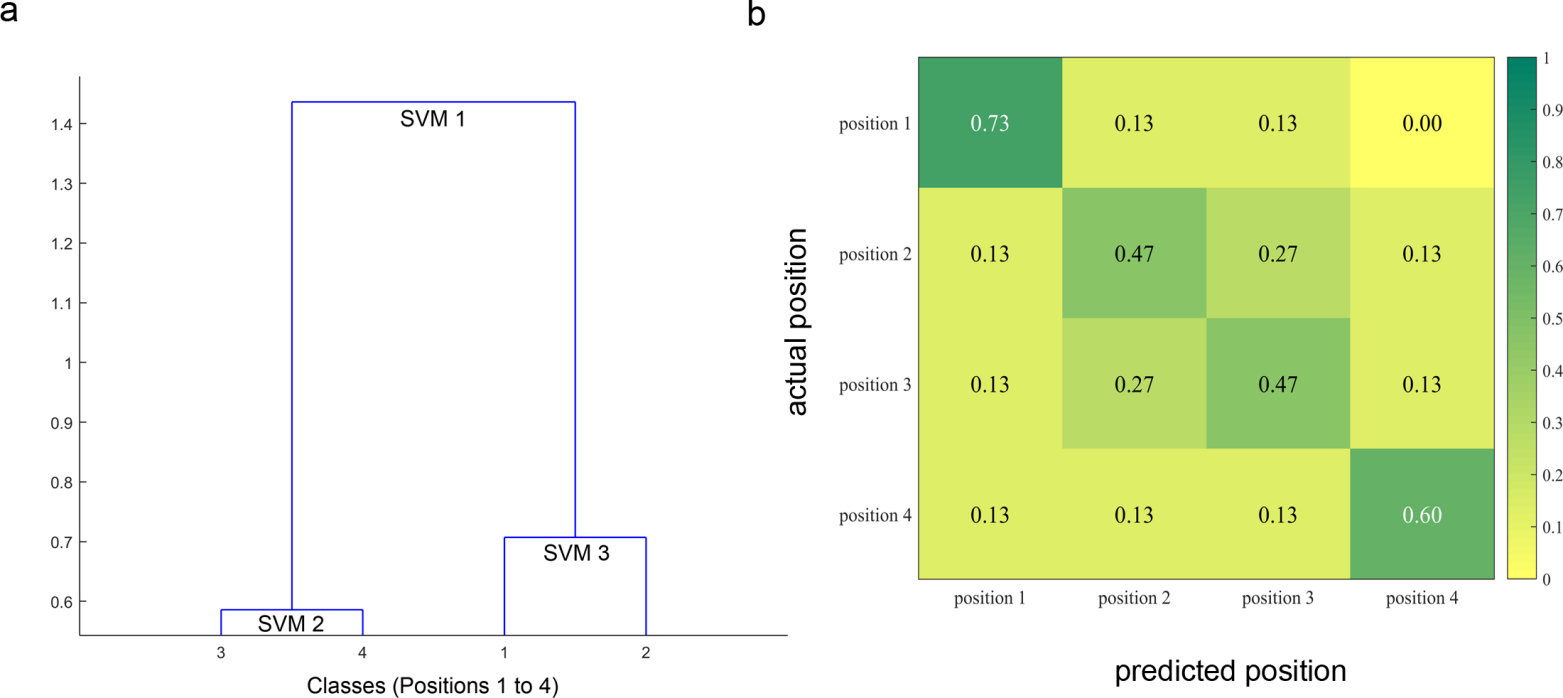
Structure of the dendrogram SVM framework and mean confusion matrix obtained with multi-feature classification. (a) Dendrogram computed via ascending hierarchical clustering (AHC) shows the multiple SVM taxonomy generated for the four classes (Locations 1 to 4) using the 12 feature space across all 15 subjects. The obtained dendrogram consists of the following 3 binary SVMs. SVM1: [Pos3/Pos4] vs. [Pos1/Pos2], SVM2: [Pos3] vs. [Pos4], and SVM3: [Pos1] vs. [Pos2] (b) Confusion matrix: Each cell shows the proportion a given actual location X (rows) that was classified as being at location Y (columns). The main diagonal therefore represents the proportion of correct classification for each of the 4 locations. Note that the mean of the main diagonal is 57% and corresponds to the overall percentage of correct classification (i.e., decoding accuracy) and that the proportions in each row sum to 1 (within rounding error in the figure), given that all events to be classified are assigned to one of four exclusive categories.

**Fig 4:**
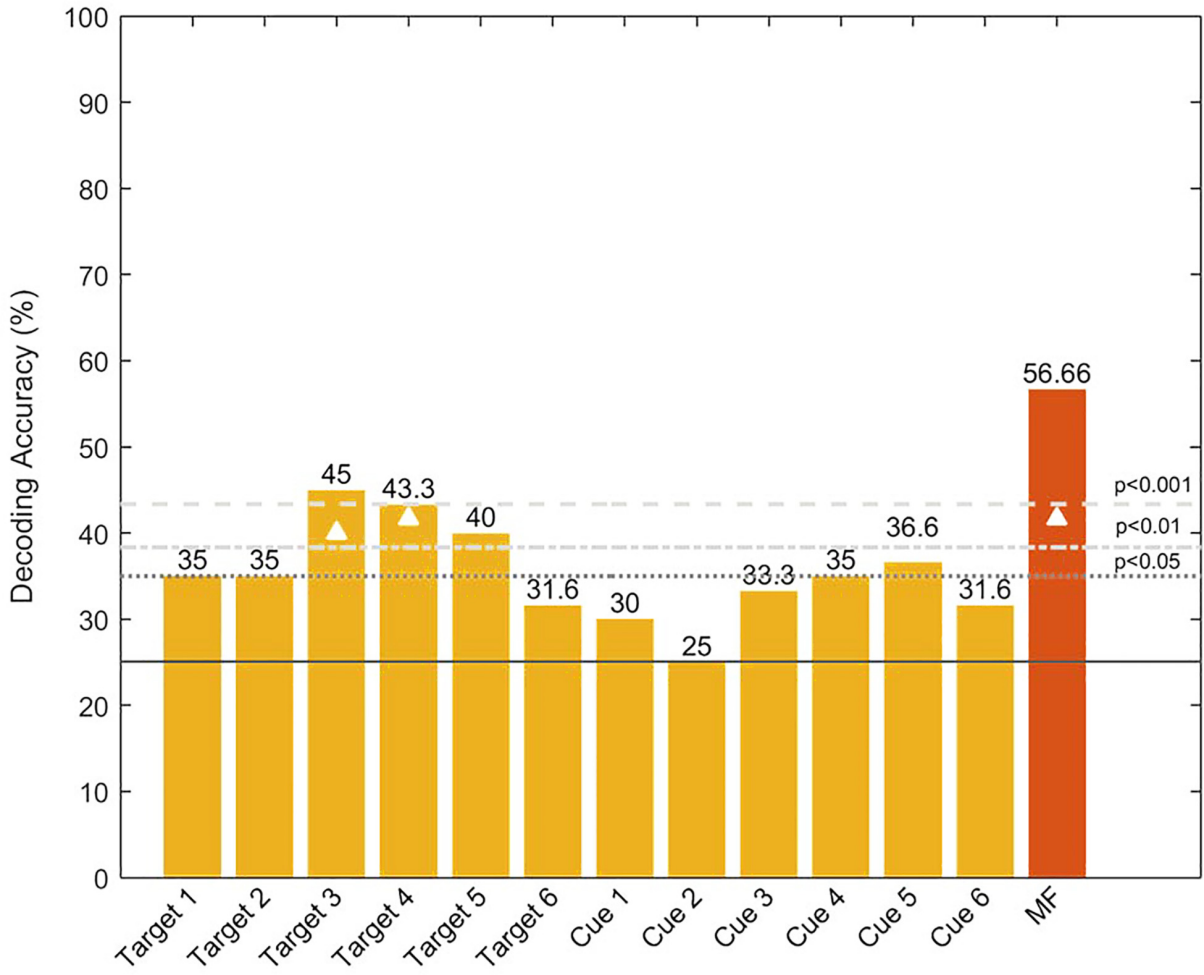
Bar chart of the single-feature decoding accuracy (DA). The features consist of the six target-locked and the six cue-locked lateralized ERPs (3 sites and 2 time-windows). The feature codes on the x-axis indicate whether they were target or cue-locked. The associated number (1–6) indicates whether a given feature was computed in the early (odd number) or late (even number) time window, and from which electrode pair it was computed (1–2: PO7/PO8, 3–4: O1/O2, and 5–6: PO3/PO4). The last (red) bar represents the multi-feature decoding accuracy obtained when we allowed the classifier to combine multiple features. The continuous horizontal line depicts the chance level (25%), while the dotted and dashed lines above it represent the statistical significance thresholds derived for the sample size using the binomial cumulative distribution. The white triangles denote the features with decoding accuracy that exceed the p < .05 level using permutation testing (Combrisson & Jerbi, 2015).

The DSVM technique yielded a 57% correct classification rate when the algorithm was allowed to combine the set of the 12 available features for cue and target locations. Fig 3 shows the confusion matrix associated with the multi-feature 4-class decoding, with columns indicating predicted locations and rows representing actual locations. Given that this is a four-class decoding problem (4 possible positions), this performance is higher than chance (25%), see also Fig 4 for statistical significance testing of the achieved decoding accuracies. The matrix shows that the outermost locations, 1 and 4, were the most easily decoded, with correct identification rates of 73% and 60%, respectively whereas the confusion rate for positions 2 and 3 was 27%. This reflects the fact that discrimination of the spatial location was more difficult when the letter was closer to the center of the display.

For each actual location, the confusion matrix (Fig 3) indicates what proportion of subjects was correctly classified, and what proportion was incorrectly classified for each of the other three classes. By combining this information with the fact that each location was separated by one letter position, we can compute the mean distance D between the actual and predicted locus of attention for each location: $D=\frac{1}{4}\sum_{i,j=1}^{4}p_{ij}|i-j|$ where *i* and *j* respectively represent the row and column indices in the confusion matrix, and *p_ij_* the probability of predicting *j* when the correct answer is *i*. On average the mean distance *D* between the predicted and the actual focus of attention was 0.62 letter positions. Given the distance to the display screen, this approximately represents a mean error of 0.55 degrees of visual angle.

The level of classification mentioned above was achieved using a multi-classification framework. In order to determine which features contributed the most to our results, we also computed their decoding accuracy. In other words, we performed 12 single-feature classifications to assess their individual decoding potential (Fig 4). Here, the probabilistic chance levels (such as 25% in a 4-class decoding problem, or 50% in a 2-class problem) are purely theoretical and are not reliable benchmarks for performance assessments in small sample sizes [36]. Due to the small sample size, we performed two additional analyses to test the statistical significance of the reported results. The significance thresholds were derived from a binomial distribution function and using permutation testing (Combrisson & Jerbi, 2015). Beyond computing decoding accuracy, additional insights into decoding precision can be gained by using measures of classification performance derived from receiver operating characteristic (ROC) curves. Typically, the area under the ROC curve (AUC) is an attractive measure as it is robust to data imbalance and does not depend on the statistical distribution of the classes. Future analyses of the data presented here may benefit from such metrics.

### Binary classification: attended versus non-attended

**Fig 5:**
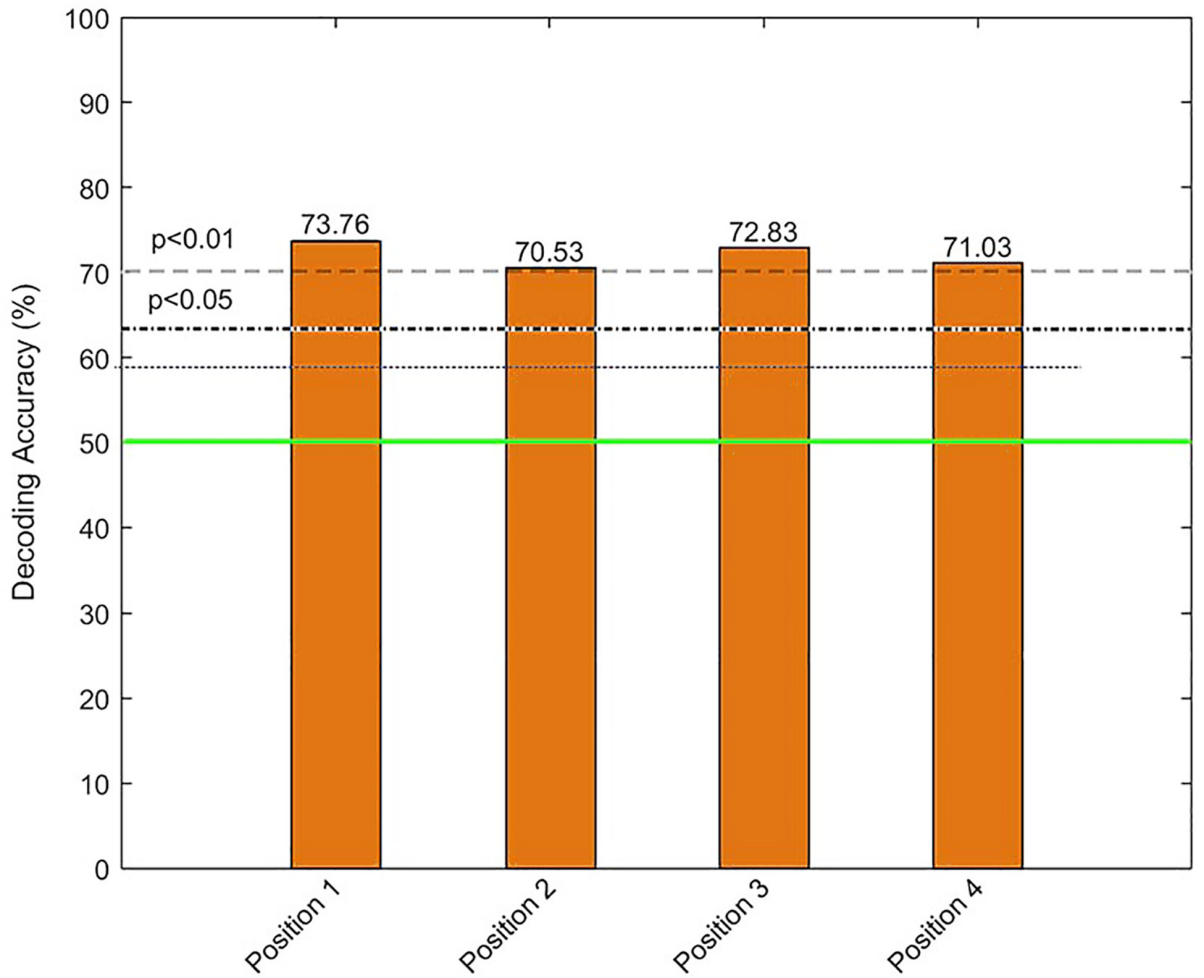
Bar chart of decoding accuracy for the binary classification—Attended vs Unattended for each of the four locations. The continuous horizontal green line represents the probabilistic chance level (50%), while the dashed and dotted lines above it represent the more reliable sample-size dependent statistical significance thresholds (at *p* < .01 and *p* < .05, respectively) using the binomial cumulative distribution. The white stars denote the features with DA that exceed the *p* < .05 level using permutation testing.

Using a similar framework, we trained a standard quadratic binary SVM (Cortes & Vapnik, 1995) to determine whether attention was deployed or not at a particular letter location at the time of its presentation. Letters were always presented at various times at each of the four sites. Thus, target-locked ERP analysis can be performed on the presentation of a letter stimulus at an attended (cued) location, or alternatively, it can be performed when time t = 0 coincides with the presentation of a letter at a non-cued location. In other words, the “attended” label corresponds to the condition for which the cue was at the same location as the target (e.g., cue at position 1, target at position 1), and the “non-attended” label corresponds to conditions for which the location of the cue does not match the position of the target (e.g., cue at position 1, target at position 2, 3 or 4). When predicting if a letter/location was attended or non-attended, the decoding accuracy achieved was statistically significant (79%, *p* < .001). We then computed the mean decoding accuracy (attended vs. non-attended) for each location separately. The results were statistically significant (*p* < .05), with a decoding accuracy above 70% for each location separately (see Fig 5).

### Ruling out confounding effects from ocular artifacts

**Fig 6:**
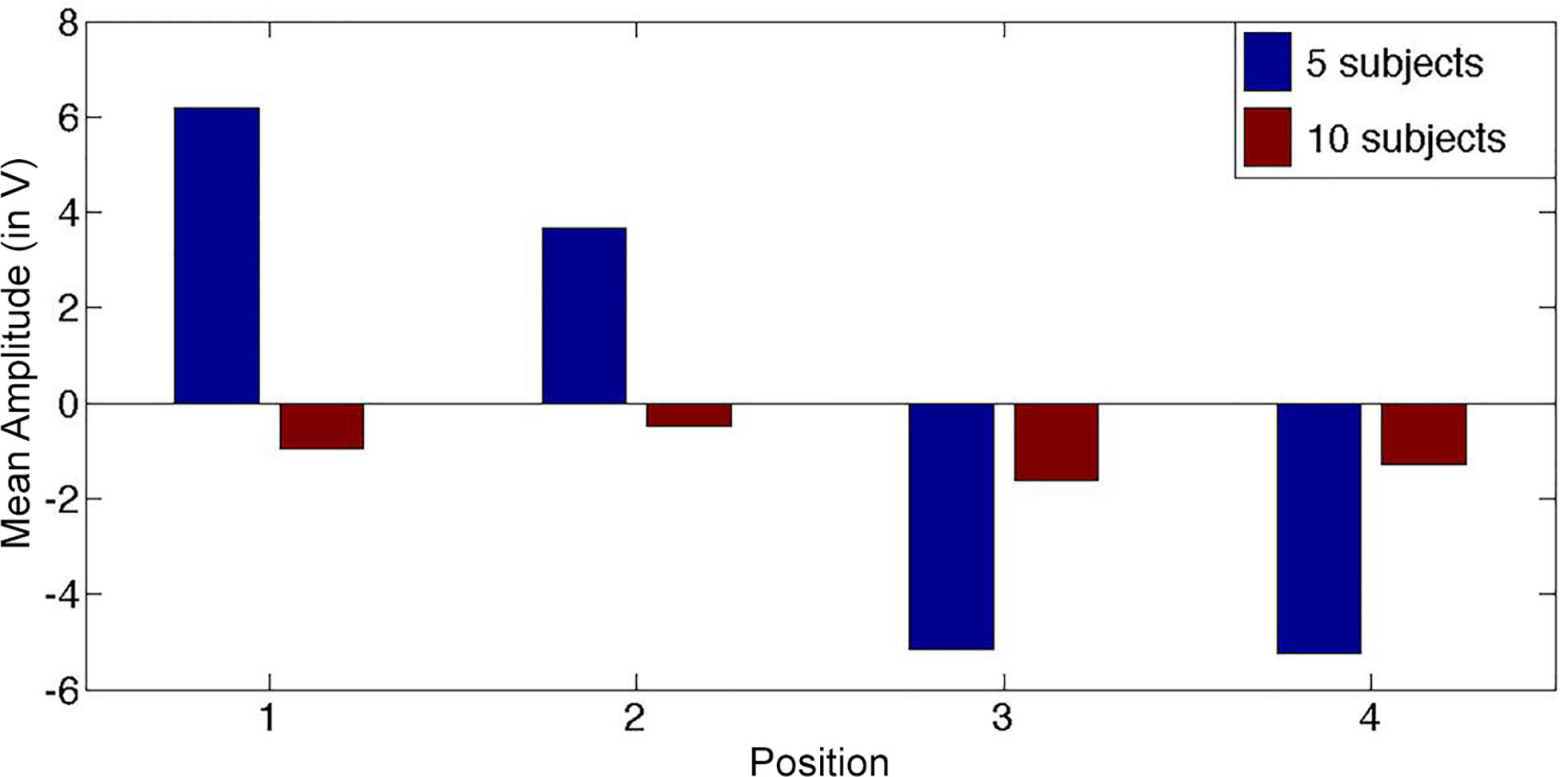
Bar plot of average HEOG amplitude at 500 ms. Blue bars represent group 1 with the 5 participants that moved their eyes the most (n = 5) and red bars represent group 2 with the 10 participants that moved their eyes the least (n = 10).

We also examined the relationship between the ERP waveforms and the EOG channels, and found that our results could not be explained by residual eye movements. We computed the lateralized HEOG1/HEOG2 waveforms and separated our total 15 participants in two groups based on the amplitude of their HEOG amplitudes (500 ms following stimulus onset): group 1 included the 5 participants that moved their eyes the most (n = 5) and group 2 included the 10 participants that moved their eyes the least (n = 10). As shown in Fig 6, we observed a larger HEOG amplitude in the waveforms computed for group 1 than in those for group 2, which reflects more eye movements.

We then applied the multi-class support vector machine (SVM) and the leave-one-out cross-validation framework to evaluate the decoding accuracy (DA) of the 10-participant data subset. The rate of correct prediction obtained was still statistically significant (DA = 50%, p < .001, permutation tests, theoretical chance level 25%). It is therefore highly unlikely that the results are affected by ocular artifacts.

## Discussion

Using a modified Posner cueing task and multi-class SVM, we investigated if lateralized ERP signals could predict the locus of visual spatial attention between four possible locations. In addition, we used the same features, to discriminate attended from non-attended stimuli. Our results showed statistically significant multi-feature decoding (*p* < .001) using ERPs recorded from occipital and parietal electrodes. This finding shows that the features we used contain class-specific information, and it provides a first step going beyond a simple left-versus-right distinction towards the decoding of a more precise locus of spatial attention using lateralized ERP features.

On the open-ended question of ‘where is attention focused?’ the accuracy of our decoder was above chance and the mean error in localizing the focus of attention was estimated at 0.55 degrees of visual angle (based on the spatial confusion matrix). Moreover, the confusion matrix revealed that locations 1 and 4 were classified with higher precision than locations 2 and 3. Given that locations 2 and 3 were closer to the fixation point at the center of the display, we deduce that there was less information regarding the lateralization of attention for locations 2 and 3. This is perhaps not surprising, given that previous work has relied on strong left-right lateralization (e.g., N2pc, SPCN), and that our stimulus locations were arrayed from left-to-right on the horizontal midline. It is not entirely surprising that the best classification was obtained for the two most extreme locations in the stimulus array. Importantly, however, we found that we could distinguish between locations within each visual hemifield, indicating that lateralized ERPs can be used to do more than just a global left-right discrimination.

By computing the decoding accuracy for each feature, we found that two features stand out (denoted as Target 03 and Target 04 in Fig 4) and give a decoding accuracy of 45% and 43.3%, respectively, which is better than chance (*p* < .001). Interestingly, these features correspond to selected time windows (0–100 ms and 410–530 ms after target onset) of the computed lateralized ERP waves at electrodes O1/O2. These results fit nicely with previous work on visual-spatial attention showing both early modulations of sensory responses by attention, as well as modulations of later components on occipital electrodes.

On the more tractable question of whether a presented stimulus is attended or not, our binary SVM classifier led to a prediction accuracy of 79% (*p* < .001). Moreover, by computing the mean decoding accuracy at each location (1, 2, 3, and 4), we showed that for each individual location we had enough information to determine whether the target was attended or not with accuracy rates of above 70%.

In this study, we focused on the feasibility of using ERPs to predict the location or presence of spatial attention. However, there are other markers of brain activity that might also be used as alternative (or complementary) features. In particular, oscillatory power modulations or long-range interactions may carry critical information with regards to the visuospatial properties of attention deployment. For instance, a previous MEG study has shown the feasibility of decoding covert attention (left vs. right hemifield) using alpha oscillations [37] and another study showed that electrocorticographic (ECoG) signals recorded from the surface of the brain provide detailed information about shifting of visual attention and its directional orientation in humans (Gunduz et al., 2012).

Decoding brain activity by using brain imaging and machine learning to predict the perceptual experience of participants has attracted attention in recent years, as it opens a range of new possibilities to determine how neural activity represents the task or stimulus information [38]. For instance, in vision, it has been successfully used to study object and face perception [39], color vision [40], and orientation processing [41]. Many other fields, such as the study of the neural processes underlying reading could also benefit from the development of this approach. The results reported in this study may be considered as another step in this direction.

Taken together, the promising findings reported here provide further confirmation that EEG signal contains discriminant features in the time-domain that can be used to decode the locus of visual spatial attention. Furthermore, using a machine learning framework, our results also support the findings of previous work on ERP components and their role in visual spatial attention processing. Although the decoding rates reported are not astounding, they are statistically significant. Moreover, we should emphasize that the displayed letters were located in relatively close proximity (about 1.15 degrees of empty space between letters). Hence, the fact that we could separate such close locations with above-chance accuracy, within visual hemifields, constitutes an important step forward for visuospatial attention decoding. We believe that the methods developed here could be further refined to infer the locus of covert attention in a number of interesting perceptual-cognitive tasks, such as during reading.

A typical ERP component that has been largely used in EEG-based signal classification frameworks is the P300 component [42,43], which is the central feature used in the P300 speller. The P300 is a well known and most widely used paradigm for the visually evoked potential brain computer interface speller, in which characters and numbers are represented in a six-by-six grid [44–46]. In a nutshell, the underlying principle is that this attention-related response is enhanced when an attended stimulus is flashed. With sufficient repetitions and a smart combination of flashing entire rows and columns of letters on a screen, the P300 wave becomes a reliable feature that can index the letter (i.e., location on a grid) that is attended. However, it has been argued that the P300-based classification paradigm relies on decoding overt spatial attention, where subjects attend to the intended letter by foveating it; that is subjects move their gaze towards the target letter (e.g., [47]. In the present study, we controlled for ocular artifacts using HEOG and VEOG channels to make sure that our signals were not contaminated by eye movements.

Future studies will be needed to increase the spatial resolution and accuracy of the decoded location of covert attention. Such studies may benefit from the inclusion of a wider range of EEG features across the spatial, temporal, and spectral domains, larger sample sizes, and other classification algorithms. As it stands, the current study can be taken as a proof of principle, showing statistically significant discrimination of the location of covert spatial attention using time-domain EEG features from parietal and occipital cortex.
